# Risk of paediatric multisystem inflammatory syndrome (PIMS-TS) during the SARS-CoV-2 alpha and delta variant waves: National observational and modelling study, 2020–21, England

**DOI:** 10.3389/fped.2022.1034280

**Published:** 2022-12-05

**Authors:** Joseph Shingleton, Lucy Burton, Hannah E. Williams, Thomas J. R. Finnie, Emma Bennett, Paul Birrell, Simon Kenny, Tiffany Watson-Koszel, Russell Viner, Moshe Arditi, Daniela DeAngelis, Nick Gent, Shamez N. Ladhani

**Affiliations:** ^1^ Emergency Preparedness Response and Resilience Directorate, UK Health Security Agency, Porton Down, London, United Kingdom; ^2^ Joint Modelling Team, UK Health Security Agency, London, United Kingdom; ^3^ MRC Biostatistics Unit, University of Cambridge, School of Clinical Medicine, Cambridge Institute of Public Health, Cambridge, United Kingdom; ^4^ Statistical Modelling and Economics, UK Health Security Agency, Colindale, United Kingdom; ^5^ CYP Transformation Programme Team, Nursing Directorate, NHS England and NHS Improvement, Leeds, England; ^6^ Institute of Systems, Molecular and Integrative Biology, University of Liverpool, Liverpool, United Kingdom; ^7^ Department of Paediatric Surgery, Alder Hey in the Park, Liverpool, United Kingdom; ^8^ Population, Policy and Practice, UCL Great Ormond St. Institute of Child Health, London, United Kingdom; ^9^ Department of Pediatrics, Division of Infectious Diseases and Immunology, Cedars-Sinai Medical Center, Los Angeles, CA, United States; ^10^ Immunisation and Countermeasures Division, UK Health Security Agency, London, United Kingdom; ^11^ Paediatric Infectious Diseases Research Group, St George’s University of London, London, United Kingdom

**Keywords:** PIMS-TS, COVID-19, SARS-CoV-2, SARS-CoV-2 alpha variant, SARS-COV-2 delta variant

## Abstract

**Objectives:**

Paediatric Multisystem Inflammatory Syndrome (PIMS-TS) is a rare life-threatening complication that typically occurs several weeks after SARS-CoV-2 infection in children and young people (CYP). We used national and regional-level data from the COVID-19 pandemic waves in England to develop a model to predict PIMS-TS cases.

**Methods:**

SARS-CoV-2 infections in CYP aged 0–15 years in England were estimated using the PHE-Cambridge real-time model. PIMS-TS cases were identified through the British Paediatric Surveillance Unit during (March-June 2020) and through Secondary Uses Services (SUS) from November 2020. A predictive model was developed to estimate PIMS-TS risk and lag times after SARS-CoV-2 infections.

**Results:**

During the Alpha wave, the model accurately predicted PIMS-TS cases (506 vs. 502 observed cases), with a median estimated risk of 0.038% (IQR, 0.037–0.041%) of paediatric SARS-CoV-2 infections. For the Delta wave, the median risk of PIMS-TS was significantly lower at 0.026% (IQR, 0.025–0.029%), with 212 observed PIMS-TS cases compared to 450 predicted by the model.

**Conclusions:**

The model accurately predicted national and regional PIMS-TS cases in CYP during the Alpha wave. PIMS-TS cases were 53% lower than predicted during the Delta wave. Further studies are needed to understand the mechanisms of the observed lower risk with the Delta variant.

## Introduction

Paediatric Multisystem Inflammatory Syndrome (PIMS-TS) is a rare life-threatening complication that typically occurs around two to six weeks after SARS-CoV-2 infection in children and young people (CYP) (1, 2). PIMS-TS shares clinical features of Kawasaki Disease (KD) and Toxic Shock Syndrome (TSS) and is characterised by single-organ or multi-organ dysfunction, with a high rate of myocardial dysfunction with cardiogenic shock, severe gastrointestinal involvement, including hyperinflammation and even subsequent transient aneurysms of the coronary arteries (2, 3).

In England, the first imported cases of SARS-CoV-2 were identified in January 2020. Endemic cases of SARS-CoV-2 increased exponentially from mid-March, reaching a peak in mid-April 2020, and then declining gradually until the end of May 2020 (4). The first reports of KD-/TSS-like presentations coincided with the first pandemic wave, initially in London, UK (3), but soon reported across England (5), and globally in countries and regions experiencing a COVID-19 pandemic wave (6–9). In response to increasing number of cases nationally, Public Health England (PHE), now known as the UK Health Security Agency (UKHSA), rapidly initiated prospective enhanced national surveillance of PIMS-TS through the British Paediatric Surveillance Unit (10).

Although the precise causal mechanisms underpinning the relationship between SARS-CoV-2 and PIMS-TS is not established, we and others have previously reported a strong temporal link between the two (10–12). During the first pandemic wave in England, both the size and timing of the peak of PIMS-TS incidence were associated with SARS-CoV-2 infection, regionally and nationally (10). In May 2020, an observational cohort study of children in Italy estimated a thirty-fold increase in the incidence of Kawasaki-like Disease during the first wave of the SARS-CoV-2 pandemic (6). In the United States, where PIMS-TS is termed multisystem inflammatory syndrome in children (MIS-C) (1, 13), the risk of PIMS-TS during the first wave of the pandemic (April to June 2020) was estimated to be 5.1 persons per million person-months or 316 persons per million SARS-CoV-2 infections in 0–20 year-olds (14). Subsequently it was noted that PIMS-TS and Kawasaki Disease had distinct characteristics and likely represented different syndromes (1, 15, 16).

Given the strong geographical and temporal relationship between SARS-CoV-2 infections and subsequent PIMS-TS risk, we postulated that it would be possible to predict, through modelling, the number of PIMS-TS cases in subsequent COVID-19 waves. This would allow for earlier planning and provision of specialist regional care services, especially cardiology and paediatric intensive care, to help improve diagnosis, investigation, and management of children with suspected PIMS-TS with the aim of improving long-term outcomes through early and effective treatment. This was particularly important in the context of a global shortage of intravenous immunoglobulin (15, 17), which remains the primary treatment for PIMS-TS along with steroids (18, 19).

Using the time lag and proportion of SARS-CoV-2 cases that resulted in PIMS-TS from previous work, we were able to provide forecasts during the *Alpha* variant wave (November 2020 to March 2021) in England. With increased testing and improved estimates of case numbers over time, along with the implementation of specific ICD-10 coding for PIMS-TS in hospital databases, we updated the model parameters to predict PIMS-TS during the *Delta* variant wave, which in England began in May 2021. Here, we report the development and execution of the model to predict PIMS-TS cases during the *Alpha* and *Delta* waves in England. We demonstrate that PIMS-TS cases during the *Delta* wave were significantly lower than predicted from cases in the previous two waves.

## Methods

### PIMS-TS hospitalisations

PIMS-TS was recognised as a new clinical syndrome following SARS-CoV-2 infection in April 2020. Because of limited data at the time, PHE initiated enhanced national surveillance through the British Paediatric Surveillance Unit (BPSU) (10). Briefly, paediatricians across the UK and Ireland are requested to electronically report cases of specific rare diseases to the BPSU every month and complete an online questionnaire about the case. From November 2020, a new emergency diagnostic code (“U07.5 Multisystem inflammatory syndrome associated with COVID-19”) was used by hospitals to record cases in Secondary Uses Services (SUS, NHS Digital, Leeds, UK), a national administrative database containing details of all admissions, emergency department attendances and outpatient appointments at NHS hospitals in England (20). Individual-level clinical and administrative data are initially collected during a patient's time at hospital and then submitted to NHS Digital for processing before being returned to healthcare providers as the Secondary Uses Service (SUS) dataset (21). We used the SUS Spell level data to identify patients with a primary or secondary (any position) U07.5 diagnosis code.

### SARS-CoV-2 incidence estimates

During the first pandemic wave, there was limited testing for SARS-CoV-2 in England and this, too, was restricted mainly to hospital settings. Children exposed to SARS-CoV-2 are typically asymptomatic or develop a mild, transient illness, such that hospitalisation for COVID-19 is rare (22). Laboratory-confirmed cases (10), or COVID-19 hospitalisation rates (8), are, therefore, likely to significantly underestimate the true rate of SARS-CoV-2 infections in children.

To overcome this issue, we used COVID-19 incidence estimates derived from the PHE-Cambridge real-time model (23, 24). This model includes inputs from the natural history of COVID-19, deaths, antibody prevalence, community infection prevalence from the Office for National Statistics' (ONS) COVID-19 Infection Survey (CIS), mean number of contacts in the population disaggregated by age-group, Google Community Mobility reports, ONS time use survey and proportion of children attending school. PIMS-TS incidence (number of cases per age-specific population) was estimated for children aged <5 years, 5–14 years and all children (<15 years). The PHE-Cambridge real-time model is continually updated to reflect the evolving landscape, including the improved understanding of natural immunity and vaccinations; the model remained stable during the period of interest in the current analysis.

### PIMS-TS prediction model

PIMS-TS cases in England identified through the BPSU were used to develop the first prediction model to forecast PIMS-TS cases during the *Alpha* wave which began in November 2020. The model assumes that a fixed proportion, ϕ, of COVID-19 infections in <15 year-olds will go on to develop PIMS-TS after a fixed lag period of τ* *days. Hence, given C(t) COVID-19 infection occurring on day t, the estimated number of associated PIMS-TS cases is estimated to be:

$$\hat{P}\left(t\right)=\phi C\left(t-\tau \right)$$

We estimate the lag parameter, τ, by applying a weekly rolling window to normalised observed weekly PIMS-TS admissions, then finding the lag-time which minimises the Root Mean Square Error (RMSE) between the normalised weekly COVID-19 infection estimates and the adjusted PIMS-TS admissions.

The estimated lag times are then used to find the optimal scaling factor, ϕ, at each week. The scaling factor is calculated in such a way as to conserve the total volume of cases observed over each *N*-week window. Hence, we can think of ϕ as the ratio of weekly PIMS-TS cases to estimated paediatric PIMS-TS infections, lagged by the time-varying lag time of τ days (see Supplementary Material for details).

The real-time model was run on a weekly basis to incorporate the most recent infection estimates in the PHE-Cambridge model. PIMS-TS estimates were, therefore, regenerated for overlapping weeks. Statistical methods employed to determine the accuracy of these early forecasts are detailed in the Supplementary Material.

The performance of the initial prediction model developed using PIMS-TS cases reported through the BPSU was assessed using PIMS-TS cases reported through SUS during the *Alpha* wave. Although predicted weekly cases matched closely with SUS-reported PIMS-TS cases, the model was re-parametrised with the new datasets at the end of the *Alpha* wave to improve case prediction in subsequent waves. This also allowed for the same data sources to be used in the model for the *Alpha* wave to be employed in subsequent variant waves for comparison.

## Results

### PIMS-TS during the first pandemic wave

Summary statistics of the parameters used to estimate PIMS-TS rates during the first pandemic wave (incidence and lag-time) are shown in Table 1 (10). During 01 March to 15 June 2020 (weeks 9–19, 2022), 268 PIMS-TS hospitalisations were reported, including 237 in children aged <15 years in England. Over the same period, the PHE-Cambridge real-time model estimated that 527,670 (95% credible interval: 396,360–759,600) SARS-CoV-2 infections in the same age cohort. PIMS-TS cases followed the epidemic curve of COVID-19 in the nine Public Health England Centre (PHEC) regions of England. In the first wave, the median lag-time between peak SARS-CoV-2 incidence and PIMS-TS incidence was 16.3 days, but with considerable variability in the lag-time parameter estimate between the nine PHEC regions (IQR, 9–24 days). Assuming that all the reported PIMS-TS cases were due to SARS-CoV-2, we estimated PIMS-TS incidence to be 45 cases per 100,000 SARS-CoV-2 infections or 0.045% (95% credible interval, 0.035% to 0.068%) in children aged <15 years.

**Table 1:** Summary statistics of the PIMS-TS and SARS-CoV-2 datasets used to estimate PIMS-TS parameters during the first wave of the COVID-19 pandemic in England.

	Any PIMS-TS case^a^ *n* (*%*)	PIMS-TS case only^b^ *n* (*%*)	SARS-CoV-2 *n* (*%*)
**Total**	268 (100%)	216 (100%)	527,670 (100%)
**Age group**			
<5 years	88 (32.8%)	73 (33.8%)	136,130 (25.8%)
5–14 years	180 (67.2%)	143 (66.2%)	391,540 (74.2%)
**Median age (IQR)**	8.2 years (4.0–12.1)	7.8 years (4.0–12.6)	

^a^ Any PIMS-TS case refers to any child fulfilling the clinical definition criteria for PIMS-TS, irrespective of whether they also fulfilled the clinical definition criteria for Kawasaki disease and/or Toxic Shock Syndrome.

^b^ PIMS-TS only cases refer to any child fulling the clinical definition criteria for PIMS-TS only, and does not fulfill the clinical definition criteria for Kawasaki disease and/or Toxic Shock Syndrome.

In children aged <5 years, the real-time model estimated 136,130 SARS-CoV-2 infections (95% CI 102,470–194,310), with an additional 391,540 (95% CI 293,890–568,290) infections in 5–14 year-olds. BPSU PIMS-TS surveillance identified 78 and 159 cases in these age groups, respectively. The estimated PIMS-TS prevalence was, therefore, 57 cases per 100,000 children aged <5 years (0.057%, 95% CI 0.040%–0.076%) and 41 cases per 100,000 5–14 year-olds (0.041%, 95% CI 0.028%–0.054%).

### PIMS-TS cases during the *alpha* wave

We used a rolling window of 8 weeks and a maximum permissible lag time of T=70 days. Supplementary Figure S1 shows the estimates for τ and ϕ over the 30-week period starting 04 January 2021. During the *Alpha* wave (weeks 1–12, 2021, when the *Alpha* variant was responsible for >90% of confirmed SARS-CoV-2 infections), the model predicted 506 (95% CI: 491–531) cases in England. This compared with 502 cases reported through SUS up to 08 March 2021 (week 10), with a median estimated lag time of 51 days (IQR 50–53 days), and a median scaling factor of 0.038% (IQR 0.037–0.041%). The estimated PIMS-TS incidence was, therefore, 38 per 100,000 SARS-CoV-2 infections in children aged <15 years.

### PIMS-TS estimates during the *Delta* wave

There were too few PIMS-TS cases between weeks 10–21 to provide reliable estimates for τ and ϕ. Between 21 May 2021 (week 20) and 02 August 2021 (week 31), when the *Delta* variant was responsible for >90% of confirmed SARS-CoV-2 infections, the median lag time increased to 62 days (IQR 61–66 days), and the median scaling factor fell to 0.026% (IQR 0.025–0.028%).

**Figure 1:**
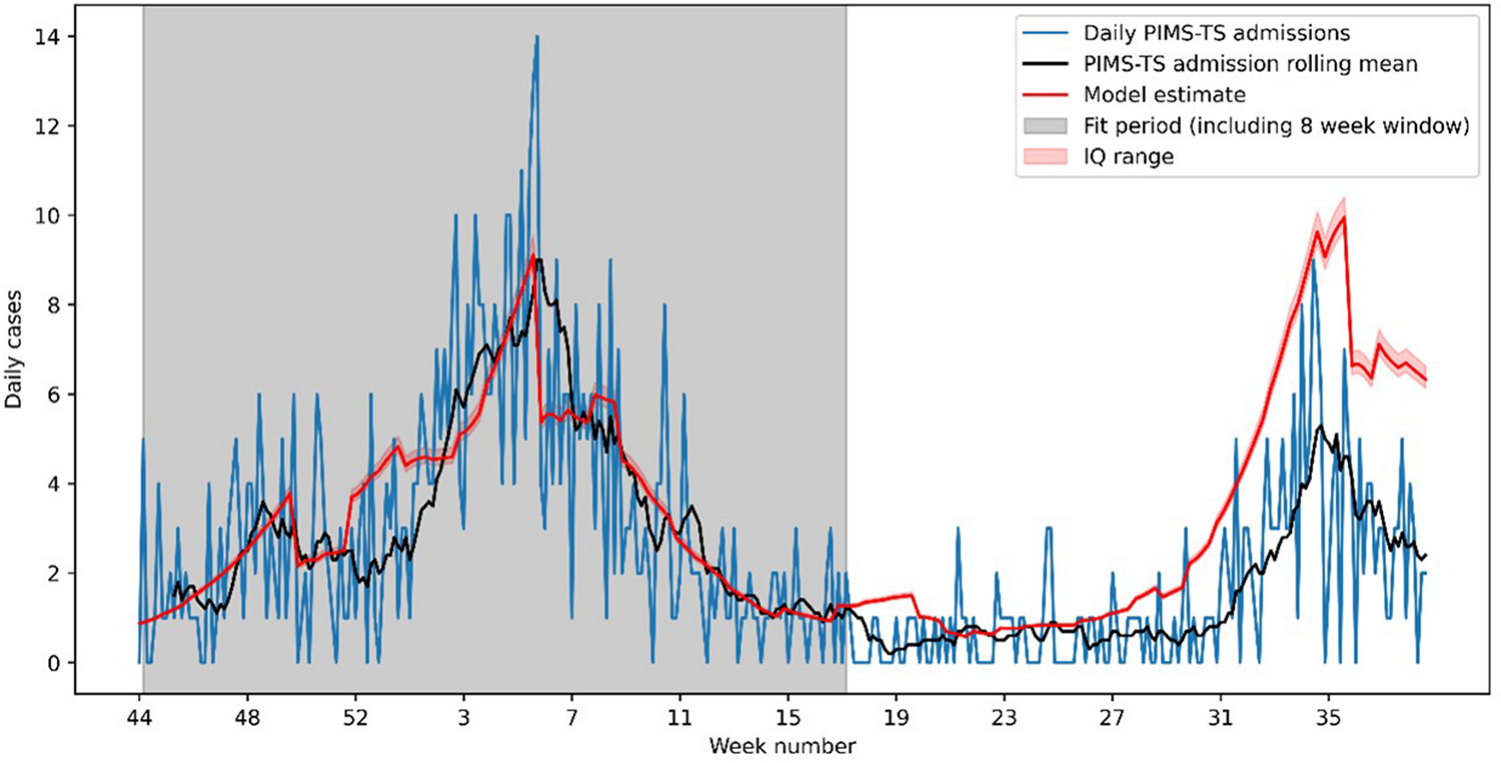
Observed and modelled PIMS-TS cases between week 44 of 2020 and week 40 of 2021. The model has been fitted to data over the *Alpha* wave (week 44 of 2020 to week 17 of 2021). The resulting model significantly underestimates the number of PIMS-TS cases in the *Delta* wave (week 27 onwards).

Figure 1 shows the estimated weekly PIMS-TS cases over this period, using the median of the parameter estimates up to week 15. The model predicted the *Alpha* wave of PIMS-TS cases well, but significantly over-estimated PIMS-TS case numbers during the *Delta* wave, with 450 (436–472) PIMS-TS cases estimated during June-October 2021 compared to 212 (53% lower) cases reported through SUS.

## Discussion

Our results confirm that PIMS-TS was rare in children and temporally and geographically associated with SARS-CoV-2 across three pandemic waves caused by the original *Wuhan* strain, the *Alpha* variant and the *Delta* variant in England. During the first pandemic wave, we estimated the risk of PIMS-TS after SARS-CoV-2 infection to be 0.045% (95% credible interval, 0.03–0.068%), which was similar to the modelled estimate of 0.038% (IQR 0.037–0.041%) for the *Alpha* wave. With the emergence of the *Delta* variant, however, PIMS-TS cases were substantially lower than the model estimates, resulting in less than half the predicted number of PIMS-TS cases.

PIMS-TS shares several clinical similarities with Kawasaki disease and toxic shock syndrome, and can manifest along a clinical spectrum of symptoms encompassing both conditions (10). A hyperinflammatory state is evidenced by clinical markers that include high serum concentrations of C-reactive protein (CRP), ferritin, and D-dimers, with increased levels of proinflammatory cytokines (5). In PIMS-TS, cardiovascular manifestations include hypotension, shock, cardiac dysfunction, myocarditis, and pericardial effusion (5).

During the first pandemic wave, we identified PIMS-TS cases through the BPSU, a well-established national system with more than three decades of experience in surveillance of rare diseases in children (10). Because of limited SARS-CoV-2 testing early in the pandemic, we used PHE-Cambridge real-time COVID-19 model outputs to estimate childhood SARS-CoV-2 infections (23). Our PIMS-TS incidence estimate of 45 cases per 100,000 children aged <15 years with SARS-CoV-2 infection is similar to the 31.6 cases per 100,000 children aged 0–20 years in the United States with the same strain and over a similar time period (14). In children aged <5 years, our estimates were 57 per 100,000 SARS-CoV-2 infections, which compares with 44/100,000 for the same age group in the United States. For comparison, the pre-pandemic incidence of Kawasaki disease in children aged <5 years was 5–15 cases per 100,000 in Europe and 19/100,000 in the United States, with rates 5–30 times higher rates in north east Asian countries such as Japan, South Korea, China, and Taiwan (1).

The mechanism by which SARS-CoV-2 triggers PIMS-TS in a minority of children remains under investigation. The delay of 2–6 weeks between infection and PIMS-TS strongly suggests a post-infectious immune mechanism. Whilst early studies identified PIMS-TS to be distinct from Kawasaki Disease and Kawasaki disease shock syndrome (KDSS) (5, 15, 16, 25), it had many more shared clinical and laboratory features with Toxic shock syndrome (26), prompting the search for a superantigen (SAg)-like motif in SARS-CoV-2 (27). Computational modeling and *in silico* studies soon identified a viral superantigen-like motif in the SARS-CoV-2 spike protein at the furin cleavage site, that can bind both MHC class II molecules and T cell receptors and may induce an inflammatory cascade, with non-specific triggering of the cytotoxic adaptive immune response (28). This motif, which is not present in other beta-coronaviruses, is highly similar in both sequence and structure to the bacterial superantigen, Staphylococcal enterotoxin B (SEB) (27), and mediates high-affinity, non-specific binding to T-cell receptors, as well as TCR skewing, which has been reported in adults with severe COVID-19, suggesting immune fingerprints of host responses to SAg (27). Specifically, children with PIMS-TS were found to have pronounced skewing and expansion of TCR Vβ repertoire in multiple studies (29–32), with up to 24% of sequenced clones expressing the TRV11–2 V region (Vβ 21.3) in one study (25). This results in massive production of pro-inflammatory cytokines from T cells and antigen-presenting cells, resulting in a cytokine storm that leads to multiorgan tissue damage, as observed in PIMS-TS, and consistent with a superantigen-driven pathogenesis (25). Recent studies have also identified an association between PIMS-TS and a combination of HLA A*02, B*35 and C*04 alleles, suggesting underlying host genetic susceptibility to PIMS-TS in CYP (31, 33), which may also explain the higher PIMS-TS rates in some ethnic groups (10, 31).

Interestingly, one study reported persistent viral reservoirs of SARS-CoV-2 in the gut of children with PIMS-TS, leading to gut dysbiosis with increased gut permeability and leakage of shed S1 spike (where the exposed SAg-like motif is located) into the circulation and correlating with severity of TCR-V11–2 skewing and serum cyotokines (34). The authors propose a role of repeated exposures to the S1 spike SAg-like motif leading to autoimmunity, in the context of viral reservoirs, in the pathogenesis of PIMS-TS as was recently suggested (35).

Finally, several immunophenotypic investigations have reported autoantibodies in children with PIMS-TS, suggesting that an autoimmune phenotype may drive this condition (25, 30, 36, 37). This is of interest because repeated exposures to viral superantigens may induce autoimmunity (38), although this is yet to be determined for SAg-like and PIMS-TS in genetically predisposed children.

Our modelling work identified a significantly lower risk of PIMS-TS during the *Delta* wave compared to the *Alpha* wave. Prior immunity to SARS-CoV-2 is unlikely to explain the lower risk because less than half the children were still infection-naïve in June/July 2021 (39), consistent with reports from other similar high income countries, and reinfection with *Delta* in previously-infected children was uncommon (40). Notably, mRNA vaccination has been shown to significantly reduce PIMS-TS risk in adolescents (41), most likely by preventing SARS-CoV-2 infection (42). In England, however, vaccination of adolescent children only began after September 2021, while vaccination of 5–11 year-olds only began after February 2022. Since each variant wave affected different geographical regions and age groups, it is possible that some of the lower risk might be due to differences in population demographics, although this alone is unlikely to explain such a large reduction in PIMS-TS risk observed with *Delta*. Additionally, given the novelty of the condition, it is possible that PIMS-TS might have been enthusiastically over-diagnosed in earlier waves especially with the broad diagnostic criteria initially developed for PIMS-TS, although it is also possible that earlier cases may have been missed because of lack of awareness of the condition. The excellent prediction of cases during the *Alpha* wave based on case numbers during the first pandemic wave however, suggests that the lower risk with the subsequent *Delta* wave is unlikely to be due to ascertainment, diagnostic or surveillance factors.

We propose that the lower risk may be explained by Spike protein mutations resulting in different amount of released S1 spike into the circulation, which in turn correlates with disease severity following SARS-CoV-2 infection (43). The furin cleavage site (PRRAR) between the S1 and S2 spike subunits plays a key role in the pathogenesis and severity of SARS-CoV-2 infection, since deletion of this cleavage site reduces S1/S2 cleavage and attenuates SARS CoV-2 pathology and virulence (44). The specific P681R Spike mutation in *Delta* that is located at the furin cleavage site between the S1 and S2 subunits at the SAg-like motif site has been associated with enhanced fitness over the *Alpha* variant (45). This mutation adds an even more polybasic residue at this cleavage site where the acidic furin cleaves the S1 and S2 subunits more efficiently. Indeed, several investigators have shown that *Delta* induces significantly more furin cleavage and more S1 products compared to *Alpha*, shedding of S1 inducing (46), as has been shown in clinical studies (47–51). We propose that a more severe acute illness with *Delta* induces more robust acute immune responses initially, which leads to fewer viral reservoirs in the gut and leakage of shed proteins into the circulation. Furthermore, this initial stronger immune response favoring more robust Th1 responses acutely would also be associated with diminished opportunity for developing subsequent autoimmunity and Th2 skewing as have been shown in other settings (52, 53).

With the recent emergence of the more transmissible *Omicron* variant, recent studies suggest an even lower risk of PIMS-TS (54, 55). Prior infection and higher vaccine uptake in adolescents will likely have contributed to this lower risk. Interestingly, though, specific Spike mutations in *Omicron*, particularly N679K, is predicted not only to induce less furin binding and cleavage and less S1 production, but may also disrupt binding of the viral SAg-motif to TCR (27), as reported in other studies (56, 57). The reason for the lower incidence of PIMS-TS during *Omicron* variant (58) may be due to significantly less S1 release and less opportunity to induce the disease to start with as well as increased protection by vaccination.

## Strengths and limitations

To our knowledge, this is the first model to predict PIMS-TS cases from community SARS-CoV-2 infection rates in real-time. We developed the model to inform regional and national healthcare prioritisation after the first pandemic wave in England. As SARS-CoV-2 testing became more widely available and healthcare databases were able to specifically code for PIMS-TS, we refined our models to provide more accurate estimates of PIMS-TS cases in subsequent waves. The strength of our model is that it uses nationally aggregated SARS-CoV-2 infection estimates and PIMS-TS hospitalisations to estimate lag period and scaling factor using a rolling window approach. This differs from previous methods for estimating lag period, which have used data disaggregated to regional level to provided static “peak-to-peak” estimates of lag time. As such, our methodology allowed us to identify parameter variation over time, at the expense of regional analysis. Whilst the estimated lag period between SARS-CoV-2 infection and PIMS-TS cases in the model was longer than previous estimates, this parameter may also be accommodating additional complexities in the whole model, which cannot be extracted from the aggregated data used to develop the model. The final model, however, was able to predict the shape and timing of weekly PIMS-TS cases, including the peak, with much greater precision during each wave.

The finding of lower PIMS-TS rates during the *Delta* wave was unexpected and could not be modelled based on previous variant waves, highlighting the limitations of modelling to predict future cases. Performing cross-country analysis, however, would allow assessment of the model's predictive accuracy in different settings and populations, and across different variant waves. The model could also be adapted to assess trends in other populations with different variants and the effects of prior infection and vaccination on PIMS-TS risks. We did not assess the clinical course, treatment or outcome of PIMS-TS across the different waves as this was outside the scope of this study.

## Conclusions

We used data from the first wave of the COVID-19 pandemic to develop and optimise a model to predict PIMS-TS cases during subsequent pandemic waves, which helped target and prioritise healthcare and treatment resources in England. The model was able to accurately predict national and regional PIMS-TS case numbers based on childhood SARS-CoV-2 infection rates during the *Alpha* wave, but PIMS-TS cases during the *Delta* wave were less than half the numbers predicted by the model. We propose that mutations leading to a differential cleavage of the SARS-CoV-2 spike protein and the resultant amounts of shed S1, which is associated with the disease severity, may explain the lower than predicted PIMS-TS cases with *Delta*, but this requires further study. Our model can be adapted for use in other settings with different variants, natural immunity levels and vaccine uptake.

## Data Availability

The original contributions presented in the study are included in the article/**Supplementary Material**, further inquiries can be directed to the corresponding author/s.

## On behalf of

BPSU PIMS-TS Investigation Team: Zahin Amin-Chowdhury, Jacob Avis, Tara Bharucha, Peter Davis, Buvana Dwarakanathan, Jessica S. Flood, Christine E. Jones, Deepthi Jyothish, Richard Lynn, Godwin Oligbu, Clare E. Pain, John Poh, Athimalaipet V. Ramanan, Mary E. Ramsay, Malcolm G. Semple, Olivia V. Swann, Elizabeth Whittaker, Christopher Williams, Rachael Wood.
