# Supplementary material for: Risk of paediatric multisystem inflammatory syndrome (PIMS-TS) during the SARS-CoV-2 alpha and delta variant waves: National observational and modelling study, 2020–21, England
Source: Front Pediatr. 2022 Dec 5;10:1034280. doi: 10.3389/fped.2022.1034280 (PMC9762156; doi:10.3389/fped.2022.1034280)

SUPPLEMENT SECTION

S1: Forecasting of the *Alpha* wave

During the early part of 2021, the parameters identified in a previous paper[10] were used to forecast PIMS-TS cases resulting from the *Alpha* wave of SARS-CoV2 infections in England. The method for producing these estimates was the same as that discussed in the main body of this paper, with an identified scaling factor and lag period applied to COVID-19 infection estimates in under-15s taken from the PHE-Cambridge COVID-19 transmission model. A total of two sets of forecasts, corresponding to two five-week periods, were provided for the *Alpha* wave. Summaries of these PIMS-TS projections are shown in Table S1.

Alpha wave forecasts of PIMS-TS cases were substantially greater than numbers of PIMS-TS cases reported in the first wave, consistent with the higher numbers of confirmed SARS-CoV-2 infections in the second wave compared to the first wave. London had the highest rates of estimated paediatric COVID-19 infections, and so correspondingly had the highest forecasted number of PIMS-TS cases.

There was a slight uplift in PIMS-TS case estimates in the second set compared to the first set of forecasts for the overlapping weeks (commencing 01 February; 08 February and 15 February) because the weekly updates in the PHE-Cambridge real-time model data included minor uplifts in COVID-19 cases (Table 2).

Table S1. Summary of the PIMS-TS projections forecasted for the second wave of the COVID-19 pandemic in England (generated from parameters estimated in the first wave)

	Expected weekly PIMS cases for weeks commencing														
	18/01/2021			25/01/2021			01/02/2021			08/02/2021			15/02/2021		
Geography	Median	5th centile	95th centile	Median	5th centile	95th centile	Median	5th centile	95th centile	Median	5th centile	95th centile	Median	5th centile	95th centile
England	57	52	62	51	45	57	44	36	56	43	31	63	45	28	75
North East	2	2	3	3	2	4	4	2	6	4	2	10	5	1	14
Yorkshire and The Humber	3	3	4	3	3	5	3	2	6	3	1	9	4	1	12
North West	8	7	9	10	8	13	12	7	20	13	6	29	15	5	40
East Midlands	4	4	5	4	3	6	4	2	7	4	2	9	3	1	11
West Midlands	6	5	7	5	4	7	4	2	7	3	1	7	3	1	8
East of England	5	4	6	3	2	3	1	1	2	1	0	1	0	0	1
London	17	14	19	12	10	15	8	5	12	5	2	10	3	1	9
South East	9	8	10	6	5	8	3	2	6	2	1	5	1	0	4
South West	3	2	3	3	3	5	4	2	6	4	2	10	4	1	14

	Expected weekly PIMS cases for week commencing														
	01/02/2021			08/02/2021			15/02/2021			22/02/2021			01/03/2021		
Geography	Value	cent5th	cent95th	Value	cent5th	cent95th	Value	cent5th	cent95th	Value	cent5th	cent95th	Value	cent5th	cent95th
England	59	54	64	46	42	51	40	35	46	38	31	45	35	27	44
North East	2	2	2	1	1	2	1	0	1	1	0	1	0	0	1
Yorkshire and The Humber	4	4	5	3	3	4	3	2	4	3	2	4	2	1	4
North West	9	8	10	6	5	7	4	3	5	3	2	4	2	1	3
East Midlands	5	4	6	5	4	6	4	3	6	4	3	7	4	2	7
West Midlands	8	7	8	8	7	9	8	6	11	9	6	13	9	5	14
East of England	7	7	8	9	8	11	11	8	14	13	9	18	13	9	20
London	13	12	15	9	7	10	6	4	7	4	3	6	3	2	4
South East	7	6	8	3	2	4	1	1	2	1	0	1	0	0	1
South West	3	3	4	2	2	3	1	1	2	1	0	1	0	0	1

S2 Statistical analyses of *Alpha* wave forecasts

Plots comparing PIMS-TS hospitalisations versus forecasts in England, by week, for the first and second set of forecasts, are shown in Figures S1 and S2, respectively, with corresponding summary statistics in Tables S2 and S3. Overall, PIMS-TS forecasts were generally in agreement with hospitalisation rates, but with some divergence, namely in week 3 (commencing 01 February 2021) and week 4 (commencing 08 February 2021). Data analysis was undertaken prior to fitting ANOVA models to ensure that model fitting assumptions held. This analysis confirmed that there were no statistically significant differences in homogeneity of variances between the PIMS-TS hospital admissions data and forecasts, where Bartlett's test $p > 0{\cdot}05$ for both sets of forecasts. These findings are evident in Figures S3 and S4, which demonstrate only minor differences between the distributions of PIMS-TS forecasts and hospitalisations by week.
The predictive accuracy of forecasts generally held when analyses were disaggregated at the regional level for the first (Figure 5) and second (Figure 6) set of forecasts, with notable exceptions in London and the North West of England. GLM fitted to check for statistically significant differences between PIMS-TS hospital admissions data and forecasts by region demonstrate excellent agreement between forecasts and hospitalisations in five of the nine PHEC regions, with no statistically significant differences observed (Table 5 and 6). There was, however, divergence the forecasts and PIMS-TS hospitalisations in the East of England, North West of England and

in London in the first set of forecasts (all $p<0{\cdot}01$) as well as in the East of England, South East of England and London in the second set of forecasts (all $p<0{\cdot}01$) (Figures 7 and 8).

Figure S1. Mean PIMS-TS admissions data versus forecasts in England (first set)

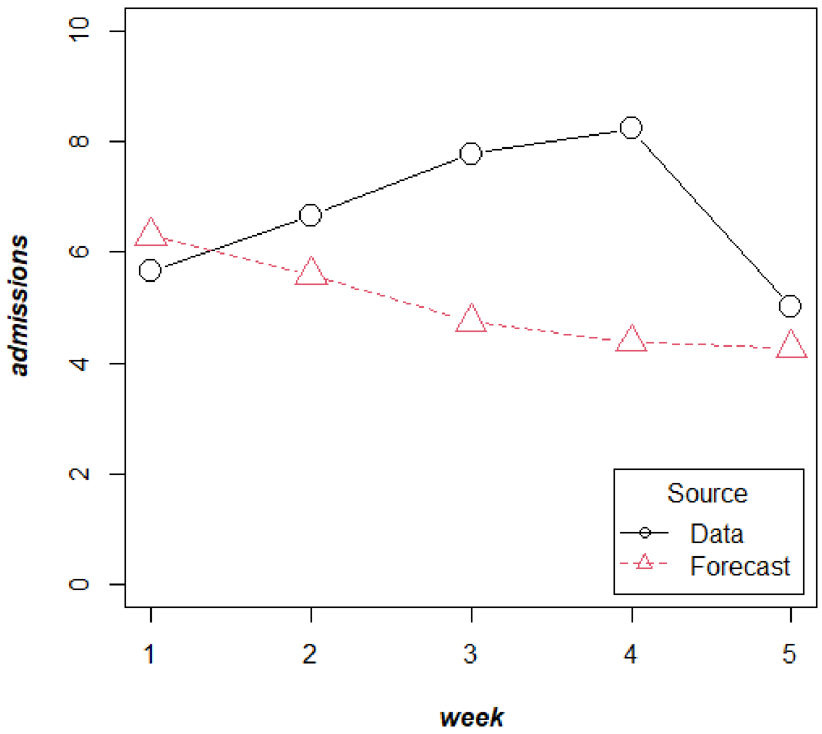

Figure S2. Mean PIMS-TS admissions data versus forecasts in England (second set)

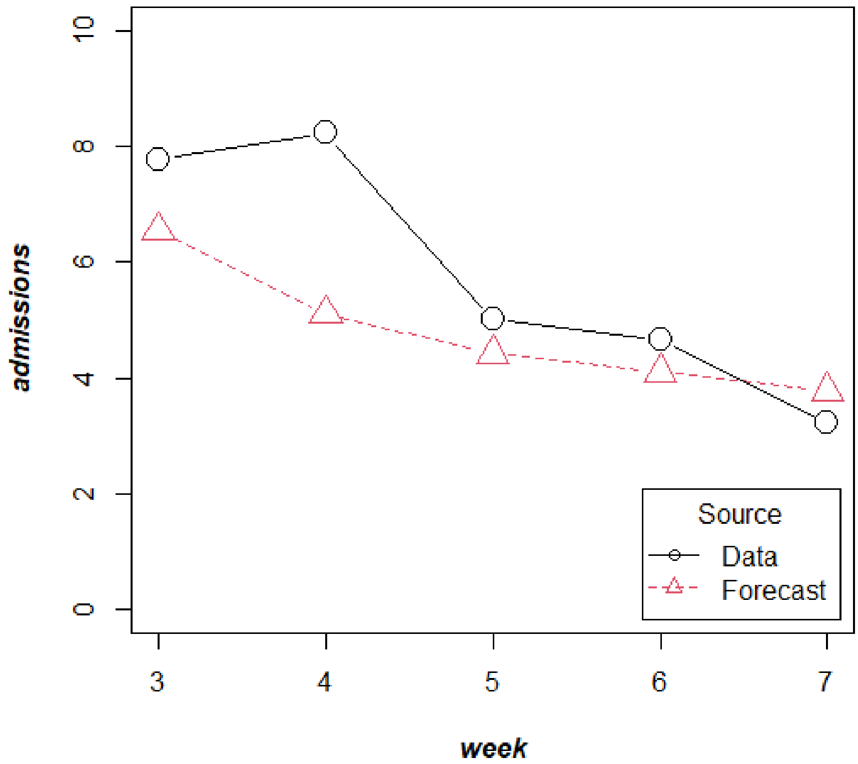

Figure S3. Boxplots of admissions data versus forecasts in England by week (first set)

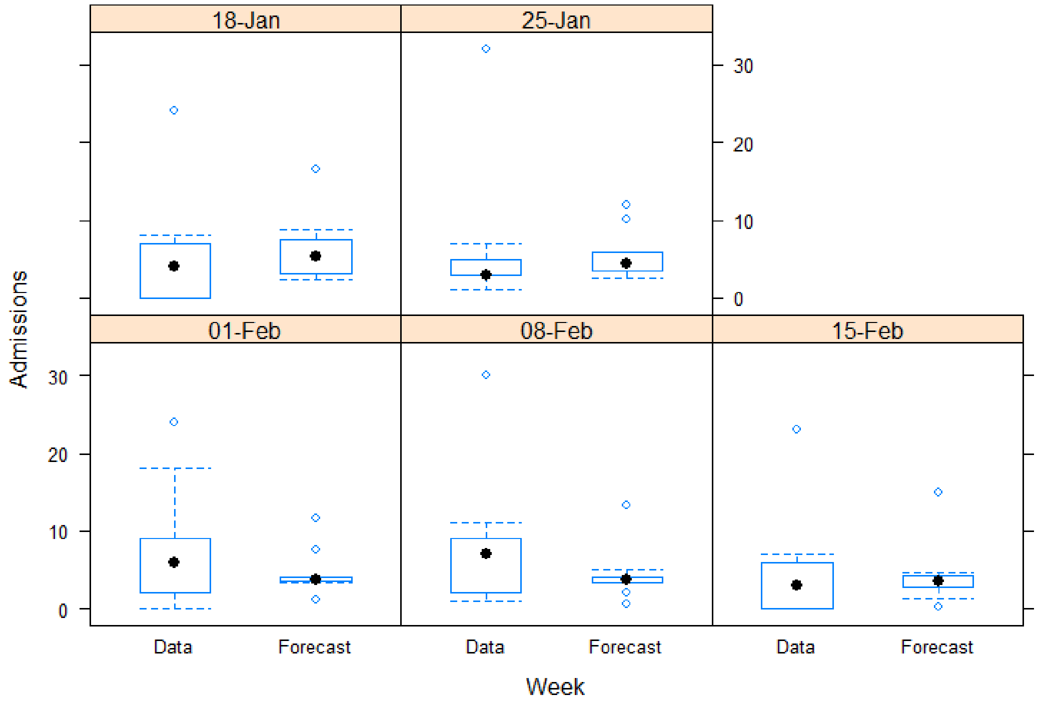

Figure S4. Boxplots of admissions data versus forecasts in England by week (second set)

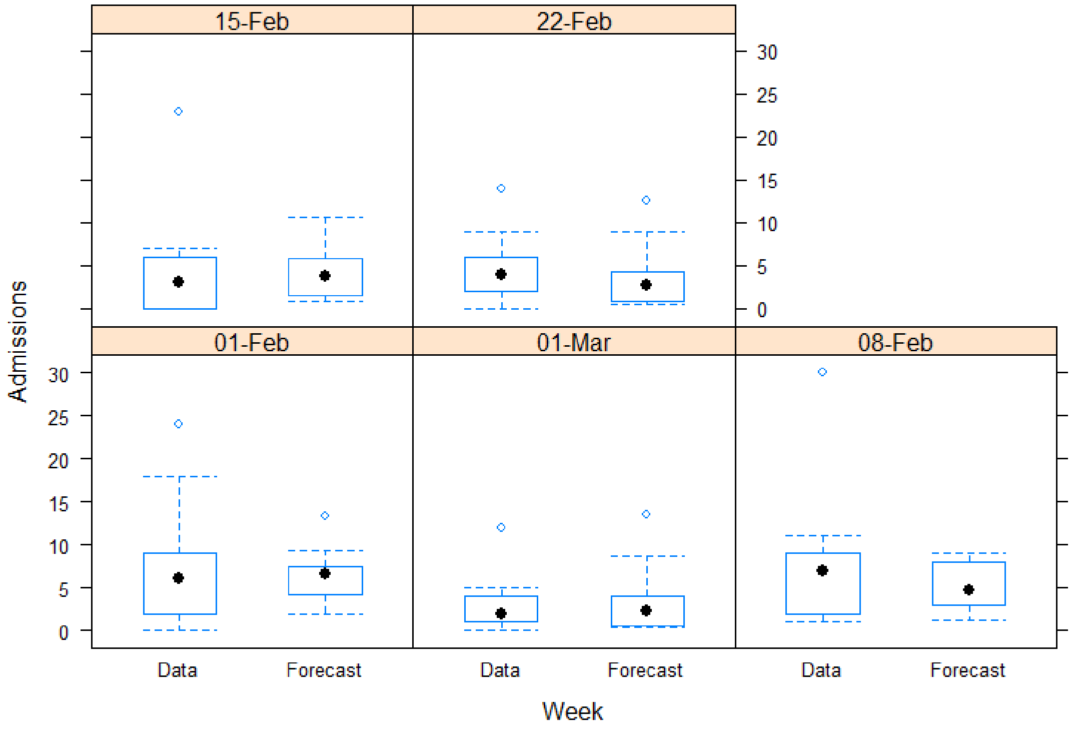

S3: Parameter Estimation using *Alpha* wave data

The *Alpha* wave of COVID-19 infections, and subsequent PIMS-TS admissions, presented an opportunity to reparameterize the model based on newly available data. As discussed in the main body of the paper, the modelling approach centres around the identification of two parameters – the scaling factor, ϕ, and the lag period, τ. Here we provide a more detailed account of how these parameters were estimated from *Alpha* wave data. We

consider a set of daily observed PIMS-TS cases, P, and a set of estimated daily paediatric COVID-19 infections, C. For a rolling window of N weeks commencing on day t, the weekly normalised PIMS-TS admissions are given by:

$$|W_p|(t_w, N) = \left\{ \sum_{d=0}^{6} |P|(t + 7n + d) \right\} \text{for } n \in \{0,1,\ldots,N\},$$

where the normalised observed daily PIMS-TS admissions, $|P|$, are given by:

$$|P| = \frac{p - P_{min}}{P_{max} - P_{min}}, \text{for } p \in P.$$

Similarly, the weekly normalised COVID-19 incidence estimate for the N-week period commencing on t_w is given by:

$$|W_c|(t_w, N) = \left\{ \sum_{d=0}^{6} |C|(t + 7n + d) \right\} \text{for } n \in \{0,1,\ldots,N\},$$

where $|C|$ is obtained through the same method used to calculate $|P|$. The lag time, τ, over the N-week period starting on day t_w is then calculated as follows:

$$\tau(t, N) = \min_{\tau \in \{0,1,\ldots,T\}} \left(\mathrm{dist}(|W_p|(t, N), |W_c|(t - \tau, N)) \right).$$

Where T denotes the maximum permissible lag-time. We use Root-Mean-Squared Error (RMSE) to calculate the distance between the normalised weekly PIMS-TS and COVID-19 cases. The RMSE at each $N = 8$ *week* period for $\tau \in \{0,1,\ldots,65\}$ days is shown in supplementary figure 1. The minimum RMSE for each week is highlighted on the plot.

Noting that $W_p(t, N)$ and $W_c(t, N)$ indicate the non-normalised weekly observed cases of PIMS-TS and paediatric COVID-19 over the N-week window starting on day t, we have:

$$\phi(t_w, N) = \frac{\sum W_p(t, N)}{\sum W_c(t - \tau(t, N), N)}.$$

Supplement Figure 2 shows the value of τ and ϕ over the 40 week period starting 1st of January 2021. These estimates use a maximum lag time $T = 65$ *days* and a rolling window of $N = 8$ *weeks*. The Python code used to estimate these parameters has been made available at [GITHUB REPO]. Please note, however, that the data is not publicly available. As such, the available code is for reference purposes only.

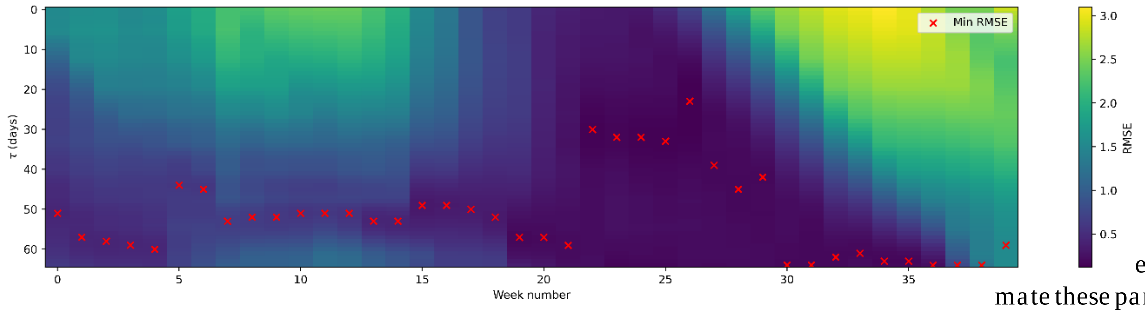

Supplement Figure 1. RMSE distance between normalised weekly COVID-19 cases and PIMS-TS cases across a range of lag times, τ, using a rolling window of $N = 8$ *weeks*.

Supplement Figure 2. Parameter estimates for lag time (τ) and scaling factor (ϕ) over the first 30 weeks of 2021.

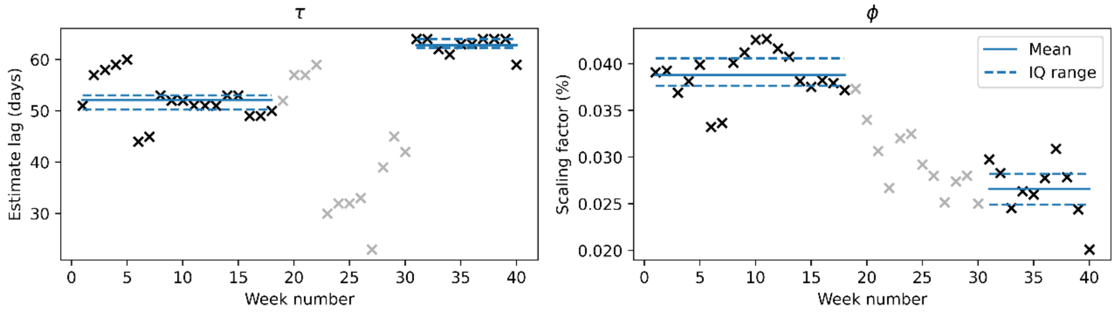

Supplement: Supplementary file 1 [file Datasheet1.pdf]
